# Prevalence of potential drug-drug interactions in Swedish pediatric outpatients

**DOI:** 10.1371/journal.pone.0220685

**Published:** 2019-08-05

**Authors:** Johan Holm, Birgit Eiermann, Elin Kimland, Buster Mannheimer

**Affiliations:** 1 Department of Laboratory Medicine, Division of Clinical Pharmacology, Karolinska Institutet, Karolinska University Hospital Huddinge, Stockholm, Sweden; 2 Inera AB, Swedish Association of Local Authorities and Regions, Stockholm, Sweden; 3 Swedish Medical Products Agency, Uppsala, Sweden; 4 Department of Clinical Science and Education at Södersjukhuset, Karolinska Institutet, Stockholm, Sweden; University of Cape Town, SOUTH AFRICA

## Abstract

**Purpose:**

To describe the occurrence of potential drug-drug interactions (DDIs) in prescribed drugs, dispensed to pediatric outpatients in Sweden.

**Methods:**

A cross sectional study was conducted based on data from a national register of prescribed drugs, dispensed at pharmacies, to children 0–17 years old. The study period was January 1 to April 30, 2010. Drug dispensing data was linked to the DDI database SFINX. Prevalence and frequencies of potential interactions were investigated, and drugs commonly involved in interactions were identified. The study focused on clinically relevant potential interactions, class D (should be avoided), and class C (can be handled, e.g. by dose adjustment).

**Results:**

In the Swedish pediatric population, 0 to 17 years of age, 12% (n = 231 078) of children had at least two dispensed drugs. In this group of patients, 0.14% had potential D-interactions and 1,3% had potential C-interactions. The number of D- and C-interactions that may lead to reduced effects were 181 (52%), and 1224 (32%) respectively. The ten most frequent drugs were involved in 78% and 65% of all potential D-, and C-interactions respectively. Furthermore, 80%, and 58% of the D-, and C-interactions respectively occurred in patients aged 12 to 17.

**Conclusions:**

We identified a limited number of drugs that were represented in the majority of potential interactions. Interactions that can lead to a reduced treatment effect constituted approximately half of D-interactions, and a third of C-interactions. The frequency of potential interactions was higher in older children. The results may contribute to increased prescriber awareness of important potential drug interactions among pediatric outpatients.

## Introduction

About one fifth of the Swedish population is younger than 18 years of age [1]. More than half of the children in Sweden receive at least one prescribed drug as outpatients annually and the rate is higher among children below the age of two years. The most commonly prescribed drugs are antibiotics followed by drugs for the respiratory tract, and dermatologicals [2]. Drug development in pediatrics has been limited and for many authorised drugs, evidence-based treatment recommendations are scarce. Consequently, off-label use of drugs in children is common, resulting in uncertainty regarding both treatment effects and adverse effects [2–5].

Exposure to an increasing number of drugs is a well-known risk factor for adverse events and has been described as a risk factor for pediatric patients in hospital settings [6, 7]. Furthermore, an increasing number of drugs has been associated with an increased risk of potential drug-drug interactions (DDIs) [8, 9], and related adverse events have been connected to potential DDIs [10]. DDIs expose patients to risks of adverse events or loss of treatment effect [11]. Consequently, awareness among prescribers of potential DDIs relevant to their patients, and knowledge of highly prevalent potential DDIs are fundamental for safe prescribing and drug use. The literature on potential DDIs in pediatric patients is relatively scarce. A few studies describe general in-, and outpatient populations in hospital settings [9, 12, 13], whereas data that focus on specific patient groups are more frequent [10, 14–19]. However, pediatric potential DDIs in the general outpatient setting, including primary health care, have not been studied in patients older than 12 months, to the best of our knowledge [20].

The aim of the present study was to describe the occurrence of potentially interacting drug combinations among all pediatric outpatients, dispensed prescription drugs at community pharmacies in Sweden.

## Materials and methods

### Study design

A retrospective cross-sectional analysis of dispensed prescription drugs was conducted, based on cohort data from the Swedish Prescribed Drug register [21]. Information on all prescription drugs in pediatric patients, aged 0 to 17 years, dispensed at Swedish pharmacies between January 1 and April 30, 2010 was retrieved from the register. All dispensed prescription drugs during the period was included. Thus, inclusion did not depend on whether drugs were reimbursed or not. Methods for analysis have been previously published in a study of patients of all ages [11]. A brief description of the method and aspects relevant to the present study, based on a pediatric subset of the whole population, is given here. The DDI-database SFINX (the name was recently changed to Janusmed interactions) [22] was used to identify potentially interacting drug combinations in the cohort. A 4-months study period allowed us to identify ongoing concomitant drug treatment based on the Swedish prescribing model, in which prescriptions for long-term treatment are renewed every three to four months. Drugs were sorted according to substance and drug form. Drug combinations that had documented risk of clinically relevant interactions in the SFINX-database were classified according to the clinical effect of the interaction, and further subcategorized in drug groups according to the Anatomical Therapeutic Chemical code (ATC classification system) [23]. In the presentation of the results, potential adverse effects of drugs that may lead to unwanted side effects were contrasted to the subcategory of adverse effects that may lead to a reduced effect of one of the interacting drugs.

Drugs were ranked according to frequency of clinically relevant potential interactions for each substance. Furthermore, interaction frequency was related to the total frequency of dispensed prescriptions for each substance during the study period. A lower cut-off level of 50, for total dispensed prescriptions during the study period, was used for inclusion on this rank-list, to avoid an overestimation of relative interaction frequency in seldom prescribed drugs.

Data on dispensed prescriptions, used to identify potentially interacting drug combinations, were included based on drug formulations relevant to the interactions occurring for each substance. In contrast, total dispensed prescription data was extracted based on ATC-codes. Total prescribing for substances with ATC-codes that do not separate relevant drug formulations from those irrelevant for interactions consequently could not be accurately calculated. However, by excluding these non-specific ATC-codes total prescribing for these substances was deliberately underestimated. Only if these substances ended up on the top list would total prescribing need to be more closely examined since their rank could only be lower than the estimated one. However, no such cases had a high enough relative frequency to be included on these lists.

### Statistical analysis

The association of clinically relevant potential DDIs with age, sex and number of prescribed drugs was analysed by unconditional binary logistic regression. We did not adjust for any other variables than these in the analysis. Associations are presented as adjusted odds ratios (OR) with 95% confidence intervals (CI). Statistical calculations were performed in R version 3.0.2 [24].

### Data sources

The Swedish Prescribed Drug Register is a register of all prescription drugs dispensed at community pharmacies in Sweden. It is maintained by the National Board of Health and Welfare and covers all prescription drugs dispensed at pharmacies to the whole population since July 2005. The register includes information on the substance and formulation of the prescribed drug, as well as age, sex, and personal identity number of the patient. The register does not include information on over the counter-drugs (OTC), drugs used in hospitals, or drugs from storage rooms in other medical institutions [21].

The SFINX database is an interaction database that has been created in collaboration between Karolinska Institutet, the Stockholm county council in Sweden, and Medbase in Finland. The name of the Swedish version of the database was changed to Janusmed interactions in 2017. The database is used in several countries and provides decision support on DDIs to prescribers. It is available through a website as well as integrated in electronic medical records systems, and it is widespread for clinical decision support on DDIs in Sweden. Interactions are classified according to clinical relevance from A to D in the database. (A) minor interaction without clinical relevance, (B) interaction with uncertain and/or variable clinical impact, (C) clinically relevant interaction that can be handled, e.g. by dose adjustments, and (D) clinically relevant interaction that is best avoided [22, 25]. More than 14 000 pairs of interacting drug combinations, documented in the database, were available for detection in the study.

### Variables

A potential DDI was defined as the occurrence of two drugs, dispensed to a patient during the study period, that interact according to the SFINX-database. Outcome variables were interactions categorized as either D-, or C-interactions. Binary variables were defined for exposure to at least one D-, or C-interaction for the regression analysis. Age categories were defined, in years, as 0–2, 3–5, 6–11, and 12–17. Categories for number of drugs were defined as 2–4, 5–7, 8–10 and 11 or more. The categories were chosen to reflect the commonly used definition of polypharmacy at >5 drugs, along with drug use immediately above and below that level. Other variables used in the analysis were patient sex and the total number of drugs prescribed to each patient during the study period. All variables were defined as categorical variables for the analysis.

### Ethical approval

The study was approved by the Regional Ethics Committee in Stockholm, Karolinska Institutet, 2008/1101–31/2. The use of prescription data in the study was also approved by the Swedish National Board of Health and Welfare that maintains the Prescribed Drug Register. Data were extracted from the register and fully anonymized before access was given to the researchers. The Regional Ethics Committee did not require informed consent to be obtained from the individuals included in the study.

## Results

The population in Sweden, 0 to 17 years of age, at the beginning of the study period was 1 921 093 people [1]. At least one dispensed drug prescription was found in 27% of the population, and 12% had at least two dispensed drug prescriptions. Total dispensing for patients with one or more prescribed drugs varied to some extent between different age groups (Table 1).

**Table 1:** Age-distribution of children in the Swedish population, and children exposed to ≥1, or ≥2 drugs.

Age group (years)	0–2	3–5	6–11	12–17	Total
**Population in Sweden**	333 152	320 652	588 201	679 088	1 921 093
**% of total**	17%	17%	31%	35%	100%
**≥1 drug**	97 414	111 879	136 595	174 475	520 363
**% of total**	19%	22%	26%	34%	100%
**≥2 drugs**	48 325	50 610	56 442	75 701	231 078
**% of total**	21%	22%	24%	33%	100%

Among patients with at least two drugs, the prevalence of potential D-interactions was 0.14% (n = 313), and the prevalence of potential C-interactions was 1,3% (n = 3 044). The number of children exposed to combinations of drugs potentially leading to D-, or C-interactions were 3 243 (1.4%), out of which 54% were girls. There were 444 (0.19%) patients with two, and 193 (0.08%) patients with three or more potential D-, or C-interactions.

Frequencies of potential DDIs, grouped according to potential clinical consequences, are shown in Tables 2–5. Tables 2 and 3 show potential D-interactions, i.e. combinations of drugs that should be avoided. The number of D-interactions that may lead to adverse effects and reduced effects were 167 (48%) and 181 (52%) respectively. Drug combinations that may lead to serotonin toxicity and/or anticholinergic side effects (n = 87) and combinations that may increase the risk of cardiac arrhythmias (n = 27) were the most common adverse effect-type potential D-interactions. The most frequent D-interactions that may lead to a reduced treatment effect involved anti-infectives (n = 73), opioids (n = 39), and contraceptives (n = 37).

**Table 2:** Frequencies of potential D-interactions with risk of toxicity, categorized according to potential clinical effect.

Potential adverse effect	Frequency
**Serotonin toxicity and/or anticholinergic side effects**	**87**
SSRI—SSRI^a^	84
Other (frequency <5)	3
**Cardiac arrhythmias**	**27**
Potassium—spironolactone/amiloride	19
Verapamil/diltiazem—beta blocker^b^	6
Other (frequency <5)	2
**Bleeding**	**24**
Warfarin—ASA low dose	14
Warfarin—metronidazole/sulfamethoxazole	6
Other (frequency <5)	4
**Sedation**	**9**
Opioid^c^ - phenobarbital	5
Other (frequency <5)	4
**Immunosuppressant toxicity**	**7**
Tacrolimus—itraconazole/posaconazole	5
Other (frequency <5)	2
**Other adverse effect (frequency < 5)**	**13**
**Total adverse effect**	**167**

Potential clinical effects and summarized frequencies in bold. For each group interaction pairs with a frequency <5 are summarized as other. Groups of potential interactions with a frequency <5 are summarized as other. SSRI selective serotonin reuptake inhibitor, ASA acetylsalicylic acid.

^a^ Sertraline, fluoxetine, paroxetine, escitalopram, citalopram.

^b^ Metoprolol, propranolol, bisoprolol.

^c^ Oxycodone, morphine, codeine.

**Table 3:** Frequencies of potential D-interactions with risk of reduced treatment effect, categorized according to potential clinical effect.

Potential reduced effect	Frequency
**Reduced anti-infective effect**	**73**
Quinolone/tetracycline^a^ - metal ion^b^	69
Other (frequency <5)	4
**Reduced analgesic effect**	**39**
Ethylmorphine—fluoxetine	18
Codeine/tramadol—fluoxetine/paroxetine	14
Other (frequency <5)	7
**Reduced contraceptive effect**	**37**
Carbamazepine—gestagen/estrogen^c^	15
Oxcarbazepine—gestagen/estrogen^d^	17
Other (frequency <5)	5
**Reduced benzodiazepine effect**	**14**
Diazepam—carbamazepine	13
Other (frequency <5)	1
**Reduced neuroleptic effect**	**10**
Risperidone—carbamazepine	8
Other (frequency <5)	2
**Other reduced effect (frequency < 5)**	**8**
**Total reduced effect**	**181**

Potential clinical effects and summarized frequencies in bold. For each group interaction pairs with a frequency <5 are summarized as other. Groups of potential interactions with a frequency <5 are summarized as other.

^a^ Ciprofloxacin, doxycycline, lymecycline, norfloxacin, tetracycline.

^b^ Calcium, magnesium, iron, aluminium, zinc.

^c^ Medroxyprogesterone, desogestrel, ethinylestradiol, drospirenone, etonogestrel, norethisterone, levonorgestrel, norelgestromin.

^d^ Medroxyprogesterone, ethinylestradiol, desogestrel, norethisterone, levonorgestrel, norelgestromin.

**Table 4:** Frequencies of potential C-interactions with risk of toxicity, categorized according to potential clinical effect.

Potential adverse effect	Frequency
**Anticonvulsant toxicity**	**982**
Valproic acid—lamotrigine	539
Valproic acid—topiramate	108
Lamotrigine—sertraline	77
Lamotrigine—carbamazepine	69
Rufinamide—valproic acid	50
Valproic acid—ethosuximide	48
Valproic acid—phenobarbital	30
Phenytoin—other anticonvulsant^a^	25
Phenytoin—diazepam	17
Gabapentin—morphine	5
Other (frequency <5)	14
**Cytostatic/immunosuppressant toxicity**	**531**
Mesalazine—azathioprine	259
Methotrexate—beta lactam antibiotic^b^	135
PPI^c^ - methotrexate/tacrolimus	94
Fluconazole—tacrolimus/sirolimus	11
Nifedipine/diltiazem—tacrolimus	10
Methotrexate—tetracycline	7
Other (frequency <5)	15
**Increased risk of bleeding**	**231**
NSAID^d^ - antidepressant^e^	179
Paracetamol–warfarin	16
Glucocorticoid^f^ - warfarin	7
ASA—antidepressant^g^	5
Levothyroxine—warfarin	6
Ethinylestradiol—warfarin	5
Other (frequency <5)	13
**Myelotoxicity**	**203**
Trimethoprim/sulfamethoxazole—methotrexate	102
Azathioprine/mercaptopurine—sulfasalazine/mesalazine/olsalazine	91
Valganciclovir—mycophenolate	8
Other (frequency <5)	2
**Acute angle closure glaucoma**	**192**
Salbutamol/terbutaline—ipratropium	192
**Neuroleptic side effects**	**84**
Fluoxetine—neuroleptic^h^	75
Other (frequency <5)	9
**Hyperkalemia and cardiac arrhythmia**	**47**
Spironolactone—ACE inhibitor/ARB^i^	47
**Serotonin toxicity**	**46**
SSRI^j^ - lithium	15
Antidepressant^k^ - tramadol	14
Sertraline—metoclopramide	6
Fluoxetine—bupropion	5
Citalopram—fluconazole^l^	5
Other (frequency <5)	1
**Increased atomoxetine levels**	**45**
Atomoxetine—fluoxetine	45
**Digoxin toxicity**	**37**
Spironolactone—digoxin	36
Other (frequency <5)	1
**Other adverse effect (frequency < 35)**	**257**
Total adverse effect	2655

Potential clinical effects and summarized frequencies in bold. For each group interaction pairs with a frequency <5 are summarized as other. Groups of potential interactions with a frequency <35 are summarized as other.

PPI proton-pump inhibitor, NSAID nonsteroidal anti-inflammatory drug, ASA acetylsalicylic acid, ACE inhibitor angiotensin-converting enzyme inhibitor, ARB angiotensin II receptor blocker, SSRI selective serotonin reuptake inhibitor.

^a^ Valproic acid, topiramate, vigabatrin, oxcarbazepine, carbamazepine.

^b^ Phenoxymethylpenicillin, amoxicillin, flucloxacillin, pivmecillinam.

^c^ Omeprazole, esomeprazole, lansoprazole, pantoprazole.

^d^ Ketoprofen, naproxen, diclofenac, ibuprofen, dexibuprofen, piroxicam, nabumetone.

^e^ Fluoxetine, citalopram, escitalopram, sertraline, paroxetine, duloxetine, venlafaxine, fluvoxamine.

^f^ Prednisolone, betamethasone, methylprednisolone.

^g^ Sertraline, fluoxetine, citalopram.

^h^ Risperidone, aripiprazole, zuclopenthixol.

^i^ Enalapril, captopril, irbesartan.

^j^ Sertraline, fluoxetine, citalopram, escitalopram.

^k^ Citalopram, sertraline, amitriptyline.

^l^ Both drugs may also increase QT-interval.

**Table 5:** Frequencies of potential C-interactions with risk of reduced treatment effect, categorized according to potential clinical effect.

Potential reduced effect	Frequency
**Reduced anticonvulsive effect**	**232**
Lamotrigine—ethinylestradiol/estradiol	42
Topiramate—oxcarbazepine	27
Clonazepam—phenobarbital	26
Topiramate—carbamazepine	25
Topiramate—phenobarbital	24
Lamotrigine—phenobarbital	20
Zonisamide—phenobarbital	11
Carbamazepine—phenobarbital	8
Valproic acid—ethinylestradiol	8
Clonazepam—phenytoin	8
Phenytoin—phenobarbital	6
Rufinamide—phenytoin	6
Carbamazepine—vigabatrin	5
Other (frequency <5)	16
**Reduced benzodiazepine effect**	**166**
Carbamazepine/phenytoin—diazepam/midazolam	164
Other (frequency <5)	2
**Reduced antihypertensive/diuretic effect**	**152**
Beta blocker^a^ - NSAID/ASA^b^	95
NSAID/ASA^c^ - diuretic^d^	35
ACE inhibitor/ARB^e^ - NSAID/ASA^f^	17
Other (frequency <5)	5
**Reduced analgesic effect**	**125**
Paracetamol—ondansetron/granisetron	91
Paracetamol–phenobarbital	17
Tramadol—ondansetron	5
Other (frequency <5)	12
**Reduced thyroid hormone effect**	**124**
Levothyroxine—metal ion/sucralfate^g^	106
Levothyroxine—ciprofloxacin	12
Other (frequency <5)	6
**Reduced cytostatic/immunosuppressant effect**	**114**
Prednisolone—tacrolimus	74
Mycophenolate—calcium/magnesium	31
Other (frequency <5)	9
**Reduced stimulation of erythropoiesis/effect on anemia**	**107**
Antacid/metal ion^h^ - iron	84
Enalapril/ramipril—recombinant erythropoietin or darbepoetin	23
**Reduced anti-infective effect**	**77**
PPI^i^ - doxycycline	48
Tetracycline—calcium/zinc	8
Calcium/magnesium—itraconazole	6
PPI^i^-itraconazole	6
Other (frequency <5)	9
**Reduced glucocorticoid effect**	**35**
Carbamazepine/phenytoin—glucocorticoid^j^	22
Phenobarbital—glucocorticoid^j^	10
Other (frequency <5)	3
**Other reduced effect (frequency < 35)**	**92**
**Total reduced effect**	**1224**

Potential clinical effects and summarized frequencies in bold. For each group interaction pairs with a frequency <5 are summarized as other. Groups of potential interactions with a frequency <35 are summarized as other.

NSAID nonsteroidal anti-inflammatory drug, ASA acetylsalicylic acid, ACE inhibitor angiotensin-converting enzyme inhibitor, ARB angiotensin II receptor blocker, PPI proton-pump inhibitor.

^a^ Propranolol, metoprolol, bisoprolol, atenolol, labetalol.

^b^ Naproxen, diclofenac, ibuprofen, ketoprofen, ASA.

^c^ Furosemide, spironolactone, bendroflumethiazide, hydrochlorothiazide, amiloride.

^d^ Diclofenac, ibuprofen, naproxen, piroxicam, ASA.

^e^ Enalapril, captopril, candesartan.

^f^ Ibuprofen, naproxen, diclofenac.

^g^ Iron, calcium, magnesium, sucralfate.

^h^ Calcium, magnesium, aluminium, sucralfate.

^i^ Omeprazole, esomeprazole, lansoprazole.

^j^ Prednisolone, betamethasone, hydrocortisone.

Frequencies of different groups of potential C-interactions, i.e. clinically relevant interactions that can be handled e.g. by dose adjustments, are shown in Tables 4 and 5. The number of C-interactions potentially leading to adverse effects and reduced effects were 2655 (68%) and 1224 (32%) respectively. Among C-interactions that may lead to an adverse effect, the most frequent types were combinations that can affect anticonvulsants (n = 982), cytostatics (n = 531), or that may cause bleeding (n = 231). Combinations that reduce the action of anticonvulsants (n = 232) and benzodiazepines (n = 166) were most frequent among C-interactions that may lead to a reduced treatment effect.

Tables 6–9 present the drugs most frequently involved in potential D-, and C-interactions. The ten most frequent drugs, in absolute numbers, were involved in 78% and 65% of all potential D-, and C-interactions respectively. The drugs that most often occurred in potential D-interactions were fluoxetine (n = 117), sertraline (n = 61) and carbamazepine (n = 42) (Table 6). However, when focusing on frequency of potential interactions (I) per number of prescribed drugs (D), warfarin and fluoxetine (I/D = 0.167 and I/D = 0.087) were most frequent followed by escitalopram (I/D = 0.081) (Table 7). The drugs most frequently involved in potential C-interactions were valproic acid (n = 797), lamotrigine (n = 750) and carbamazepine (n = 391) (Table 8). When relating potential interaction frequency to the total volume of prescribed drugs, phenobarbital and azathioprine were the drugs most commonly involved (I/D = 0.748 and I/D = 0.65) (Table 9).

**Table 6:** Frequency, in absolute numbers, of the 10 most common drugs involved in potential D-interactions.

Rank	Drug	Interaction frequency
1.	Fluoxetine	117
2.	Sertraline	61
3.	Carbamazepine	42
4.	Iron	39
5.	Ciprofloxacin	33
6.	Warfarin	24
7.	Calcium	22
8.	Lymecycline	20
9.	Ethylmorphine	19
10.	Potassium	19
…		
18.	Escitalopram	12

Only drugs with total prescribing of at least 50 were included in the table. The rank of the top 3 drugs in Table 7 are given at the bottom of Table 6 if they are not among the top ten in this table.

**Table 7:** Frequency, in relation to the total number of dispensed drugs, of the 10 most common drugs involved in potential D-interactions.

Rank	Drug	n interactions/ n dispensed drugs
1.	Warfarin	0,167
2.	Fluoxetine	0,087
3.	Escitalopram	0,081
4.	Spironolactone	0,076
5.	Phenobarbital	0,048
6.	Carbamazepine	0,045
7.	Ciprofloxacin	0,044
8.	Venlafaxine	0,039
9.	Citalopram	0,03
10.	Medroxyprogesterone	0,027
…		
12.	Sertraline	0,023

Only drugs with total prescribing of at least 50 were included in the table. The rank of the top 3 drugs in Table 6 are given at the bottom of Table 7 if they are not among the top ten in this table.

**Table 8:** Frequency, in absolute numbers, of the 10 most common drugs involved in potential C-interactions.

Rank	Drug	Interaction frequency
1.	Valproic acid	797
2.	Lamotrigine	750
3.	Carbamazepine	391
4.	Azathioprine	336
5.	Methotrexate	293
6.	Mesalazine	270
7.	Fluoxetine	213
8.	Ipratropium	192
9.	Sertraline	191
10.	Topiramate	190
…		
13.	Phenobarbital	157
32.	Cyclosporine	59

Only drugs with total prescribing of at least 50 were included in the table. The rank of the top 3 drugs in Table 9 are given at the bottom of Table 8 if they are not among the top ten in this table.

**Table 9:** Frequency, in relation to the total number of dispensed drugs, of the 10 most common drugs involved in potential C-interactions.

Rank	Drug	n interactions/ n dispensed drugs
1.	Phenobarbital	0,748
2.	Azathioprine	0,65
3.	Cyclosporine	0,596
4.	Ipratropium	0,565
5.	Rufinamide	0,56
6.	Ethosuximide	0,495
7.	Topiramate	0,49
8.	Spironolactone	0,441
9.	Carbamazepine	0,423
10.	Mesalazine	0,412
…		
15.	Valproic acid	0,321
17.	Lamotrigine	0,292

Only drugs with total prescribing of at least 50 were included in the table. The rank of the top 3 drugs in Table 8 are given at the bottom of Table 9 if they are not among the top ten in this table.

The frequency of potential DDIs in different age groups are presented in Tables 10 and 11. Patients aged 12 to 17 contributed with 80% of the potential D-interactions (278/348) (Table 10). The corresponding proportion of potential C-interactions in the same age group was 58% (2246/3879) (Table 11). In this age group, the most common types of potential D-interactions were those attributed to serotonin toxicity and/or anticholinergic side effects (n = 84). Among D-interactions that may lead to a reduced treatment effect, anti-infectives (n = 67) and analgesics (n = 37) were most common. Among 2246 potential type C-interactions in the same age group, combinations that may lead to adverse effects from anticonvulsants (n = 507), cytostatics or immunosuppressants (n = 359), or that may cause bleeding (n = 209) were most frequent. Common C-interactions that may lead to a reduced treatment effect involved anticonvulsants (n = 114) and antihypertensive or diuretic drugs (n = 104). With very few exceptions, the highest number of potential interactions for each type occurred in the oldest age group.

**Table 10:** Frequencies of D-interactions with various potential clinical effect in different age groups.

Age group (years)	0–2	3–5	6–11	12–17	Total
**Potential adverse effect**	**13**	**15**	**20**	**119**	**167**
Serotonin toxicity and/or anticholinergic side effects	0	0	3	84	87
Cardiac arrythmias	10	1	4	12	27
Bleeding	1	10	4	9	24
Sedation	0	1	6	2	9
Immunosuppressant toxicity	0	2	0	5	7
Other adverse effect (frequency <5)	2	1	3	7	13
**Potential reduced effect**	**3**	**2**	**17**	**159**	**181**
Reduced anti infective effect	0	0	6	67	73
Reduced analgesic effect	0	1	1	37	39
Reduced contraceptive effect	0	0	3	34	37
Reduced benzodiazepine effect	2	1	3	8	14
Reduced neuroleptic effect	0	0	2	8	10
Other reduced effect (frequency <5)	1	0	2	5	8
**Total**	**16**	**17**	**37**	**278**	**348**

**Table 11:** Frequencies of C-interactions with various potential clinical effect in different age groups.

Age group (years)	0–2	3–5	6–11	12–17	Total
**Potential adverse effect**	**122**	**322**	**657**	**1554**	**2655**
Anticonvulsant toxicity	28	113	334	507	982
Cytostatic/immunosuppressant toxicity	10	59	103	359	531
Increased risk of bleeding	3	6	13	209	231
Myelotoxicity	3	62	47	91	203
Acute angle closure glaucoma	31	41	59	61	192
Neuroleptic side effects	0	1	10	73	84
Hyperkalemia and cardiac arrhythmia	15	4	11	17	47
Serotonin toxicity	0	0	1	45	46
Increased atomoxetine levels	0	0	7	38	45
Digoxin toxicity	16	5	9	7	37
Other adverse effect (frequency <35)	16	31	63	147	257
**Potential reduced effect**	**89**	**140**	**303**	**692**	**1224**
Reduced anticonvulsive effect	33	28	57	114	232
Reduced bensodiazepine effect	11	37	60	58	166
Reduced antihypertensive/diuretic effect	15	5	28	104	152
Reduced analgesic effect	5	23	36	61	125
Reduced thyroid hormone effect	7	8	20	89	124
Reduced cytostatic/immunosuppressant effect	6	15	28	65	114
Reduced stimulation of erythropoiesis/effect on anemia	4	8	23	72	107
Reduced antiinfective effect	0	3	8	66	77
Reduced glucocorticoid effect	5	4	12	14	35
Other reduced effect (frequency <35)	3	9	31	49	92
**Total**	**211**	**462**	**960**	**2246**	**3879**

The association between the variables age, sex, number of drugs, and one or more potential D-, or C-interaction was analysed. Boys aged 0 to 2 years with two to four drugs were used as reference category for the analysis. Adjusted odds ratio (OR) for girls was 1.10 (95% CI 1.02, 1.18). For age categories 3 to 5, 6 to 11, and 12 to 17, odds ratios were 2.10 (95% CI 1.74, 2.53), 3.74 (95% CI 3.16, 4.45), and 6.89 (95% CI 5.88, 8.13), respectively. For categories of increasing number of drugs, odds ratios were 6.46 (95% CI 5.95, 7.00) with five to seven drugs, 21.14 (95% CI 18.78, 23.75) with eight to ten drugs, and 59.65 (95% CI 50.90, 69.79) with more than eleven drugs.

## Discussion

### Results discussion

To our knowledge, this nationwide study is the first reporting on potential drug-drug interactions in general pediatric outpatients, 0 to 17 years. Most previous studies of prevalence of potential interactions among pediatric patients have been limited to hospital settings [9, 12] or specific patient groups [10, 14–19]. Only one study has previously been published on general pediatric outpatients less than one year old [20].

We found potential D-, or C-interactions in 1.4% of outpatients, exposed to at least two drugs. This low prevalence probably illustrates the relatively healthy general outpatient population compared to populations in hospital settings. In a retrospective cohort study of 498 956 hospitalizations of <21-year-old patients, conducted in the USA, 49% of hospitalizations were associated with potential DDIs. Only 5% of hospitalizations were associated with a contraindication, the most serious potential interaction class, whereas 41% were associated with a major potential DDI [12]. In contrast, another retrospective study of 6078 outpatients aged 0 to 19 years, from a single hospital in the Czech Republic, identified potential DDIs in only 3.83% of patients, with moderate to severe cases in 0.47% [9]. Different DDI databases were used to identify cases in the two studies, and the American study included only hospitalized patients whereas the Czech study included outpatients at the hospital. Furthermore, patients in the American study were older. These factors may indicate why percentages of exposure to potential DDIs differed so much between the two studies.

In the present study, interactions that may lead to a reduced treatment effect constituted 52%, and 32% of D-, and C-interactions respectively. Evidence suggest that this type of interactions may be overlooked as compared to interactions leading to overt adverse drug effects and may therefore be particularly important to acknowledge [26, 27]. In our investigation, the majority of these potential interactions resulted from combinations of drugs containing metal ions that inhibit the absorption of antibiotics, analgesic ‘prodrugs’ combined with drugs impairing their bioactivation, and potential interactions involving antiepileptics leading to an increased metabolism of co-dispensed drugs (Tables 2–5). Previous studies of prevalence of potential DDIs in pediatric patients lack this dimension. However, results from our previous study, including the whole Swedish population, were in line with the present study of the pediatric patients. In our previous study, approximately half of all potential interactions were combinations that may lead to a reduced treatment effect [11].

Furthermore, we listed the top ten drugs involved in potential DDIs for D- and C-interactions respectively. The drugs on these lists were involved in 78% of D-, and 65% of C-interactions. Attention to potential DDIs with these relatively few drugs may help improve patient safety considerably. Similar to our findings, in an American hospital setting opioids, anti-infective, neurologic, gastrointestinal, and cardiovascular agents were commonly involved in potential interactions [12]. These drug-groups remained frequent in our top-ten lists when frequencies of potential interactions were related to total prescribing of each substance. We also identified immunosuppressants among the most common drugs involved in potential DDIs. Furthermore, the Czech study also conducted in a hospital setting, describe an association between immunosuppressants, antimycotics, antiepileptic, antineoplastic agents, drugs for obstructive airway diseases, and increased odds for a potential DDI [9]. Contrary to our findings, with ciprofloxacin and lymecycline on the top-ten-list, and with tetracyclines and quinolones involved in common D-interactions, antibiotics for systemic use were not associated with significantly increased odds of potential DDIs in their analysis.

Children aged 12 to 17, contributed with four fifths of potential D-interactions and more than half of potential C-interactions. The number of patients with dispensed drugs varied slightly between different age groups but not to the extent that exposure to interactions did. In fact, older age was associated with an increased risk of exposure to a potential D-, or C-interaction with the highest odds ratio among patients 12 to 17 years old. This association was independent of the number of dispensed drugs and it is likely that frequencies of potential interactions vary due to variations in the type of drugs prescribed to different age groups.

### Strengths and limitations

The Prescribed Drug Register covers all community pharmacies in Sweden and the use of a nationwide cohort, with close to complete coverage of the outpatient drug use, is an important strength of this study. Drugs dispensed at hospitals are not included in the register and the aim of this study has been to describe potential DDIs in outpatients. However, use of OTC-drugs are not included in the Prescribed Drug Register and could not be included in the analysis. Commonly used OTC-drugs include NSAIDs, acetylsalicylic acid, and paracetamol as well as antacids, and proton pump inhibitors. All of these drugs were represented among potential interactions in the analysis. Consequentially, frequencies of these potential interactions can be expected to be underestimated.

Co-treatment with interacting drugs was defined as dispensing of two drugs within the same four-month interval. This definition was based on the Swedish prescribing model, in which prescriptions are renewed every three to four months. A potential drawback of this strategy is that it may lead to an overestimation of interactions by defining changes in drug treatment, between drugs that are not used at the same time, as co-treatment. It is not unlikely that e.g. simultaneous treatment with two SSRIs is to some extent due to this effect rather than actual co-treatment. Another type of potential interactions that may be overestimated are those that may be handled by a temporary brake in the treatment while an interacting drug is given. E.g. drugs that are not used during a short treatment with an antibiotic.

An important strength of the study design is the general perspective that is given on potential DDIs in the pediatric population. However, with the broad perspective provided, some potential interactions may be detected that cannot be described in enough detail to evaluate the clinical relevance. The combination of methotrexate and a PPI for example, is only relevant in patients receiving high dose methotrexate. Another example is the combination of salbutamol/terbutaline and ipratropium that is relevant only if the drugs are concomitantly administered with a nebulizer.

The DDI database SFINX has become widely used in Sweden since it was introduced in the country in 2007, and it is now integrated into almost all medical records systems. It was utilized in health care and contained more than 14 000 pairs of interacting drugs at the time of the study, and it is regularly updated. The database can therefore be considered a good approximation of the information that prescribers have at their disposal at the time of prescribing. Since the study period, the interaction database has expanded, and more interactions and new drugs are continuously added. However, the interactions detected are based on a previous version of the database, relevant to the time of the study period. Furthermore, new drugs have been registered and prescribing guidelines may have changed. Consequentially, the frequencies of specific drugs and interactions may have changed to some extent, and results should be interpreted with this in mind.

A limitation of the database is the distinction between category D-, and C-interactions. By definition, D-interactions should be avoided whereas C-interactions can be handled by for instance dose adjustment. In reality, some interactions classified in category D can actually be handled as well. E.g. combinations of tetracyclines or quinolones with metal ion-containing drugs, where separation of the administration allows for co-treatment without reduced effect [28]. Another limitation of the DDI database, with regards to pediatric patients, is that evidence for the interaction effects are not generally based on studies in children. Consequentially, the clinical relevance of interactions may differ in children compared to what is established for adults.

## Conclusion

This nationwide study identifies common potential drug interactions among pediatric outpatients. We identified a limited number of drugs that were represented in a major part of interactions. Frequent type D-interactions involved drug combinations that may cause serotonin toxicity and/or anticholinergic side effects, increase the risk of cardiac arrhythmias, or lead to a reduced treatment effect of anti-infectives, opioids, or contraceptives. Among type C-interactions, combinations that can affect anticonvulsants, cytostatics, or that may cause bleeding, or reduce the effect of anticonvulsants or benzodiazepines were common. Interactions that can lead to a reduced treatment effect constituted approximately half of D-interactions and a third of C-interactions. This type of interactions may be overlooked, and prescribers need to be aware of the high percentage of interactions in clinical practice that this group constitutes. Interaction frequency was higher in older children. The results of this study may contribute to increased awareness of clinically important drug interactions in the pediatric population among prescribers.
